# Supplementary material for: Superconducting metamaterials for waveguide quantum electrodynamics
Source: Nat Commun. 2018 Sep 12;9:3706. doi: 10.1038/s41467-018-06142-z (PMC6135821; doi:10.1038/s41467-018-06142-z)
Supplement: Supplementary file 1 — Supplementary Information [file 41467_2018_6142_MOESM1_ESM.pdf]

Supplementary Information for: "Superconducting metamaterials for waveguide quantum electrodynamics"

Mohammad Mirhosseini,[1,2] Eunjong Kim,[1,2] Vinicius S. Ferreira,[1,2]
Mahmoud Kalaee,[1,2] Alp Sipahigil,[1,2] Andrew J. Keller,[1,2] and Oskar Painter[1,2,*]

[1] *Kavli Nanoscience Institute and Thomas J. Watson, Sr., Laboratory of Applied Physics, California Institute of Technology, Pasadena, California 91125, USA.*

[2] *Institute for Quantum Information and Matter, California Institute of Technology, Pasadena, California 91125, USA.*

(Dated: August 1, 2018)

* opainter@caltech.edu; http://copilot.caltech.edu

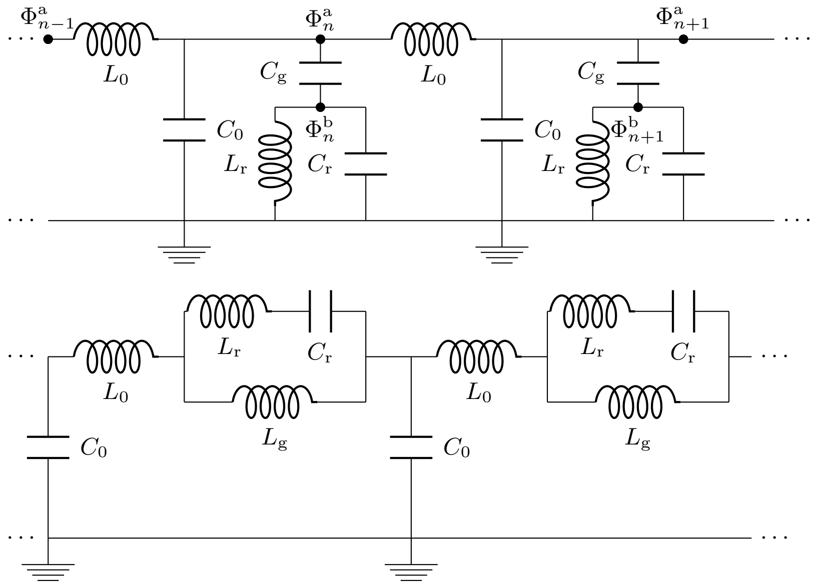

Supplementary Figure. 1. **Circuit diagram of metamaterial waveguide.** The waveguide can be made from periodic arrays of transmission line sections loaded with capacitively coupled resonators (top), or inductively loaded resonators (bottom).

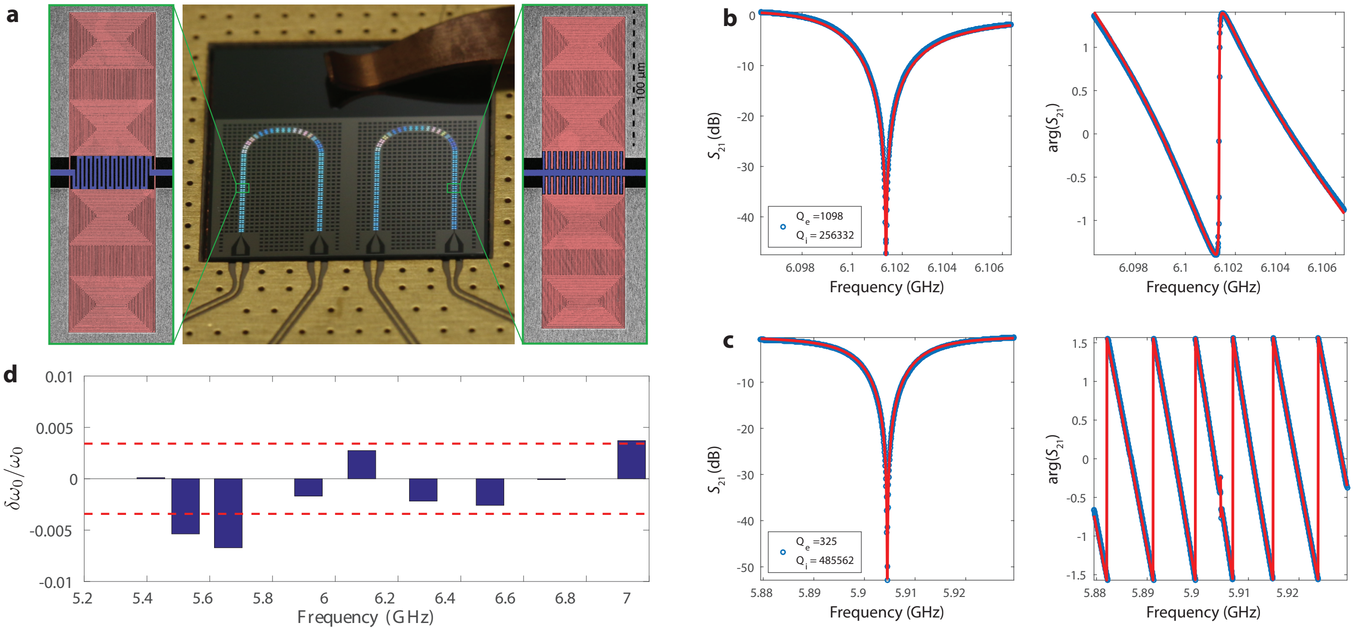

Supplementary Figure. 2. **Characterization of lumped element resonators a**, Optical and SEM images of microwave resonator array chip. Middle: optical image of the chip with two arrays of coupled resonators on a 1×1 cm silicon chip. Left and Right: SEM image (false-color) of the fabricated inductively (left) and capacitively (right) coupled microwave resonator pairs. The resonator region is colored red and the waveguide central conductor is colored blue. **b-c**, Amplitude and phase response of two capacitively-coupled microwave resonator pairs measured at the fridge temperature $T_f \approx 7$ mK. The legends show the intrinsic ($Q_\mathrm{i} = \omega_0/\gamma_\mathrm{i}$) and extrinsic ($Q_\mathrm{e} = \omega_0/\gamma_\mathrm{e}$) quality factors extracted from a Fano line shape fit. **d**, Difference between the measured and the expected design value of the resonance frequencies for 9 resonators with similar geometries and wire widths of 500 nm. The dashed lines mark the standard deviation of the frequency difference, which is equivalent to a normalized value of $\sigma = 0.3\%$.

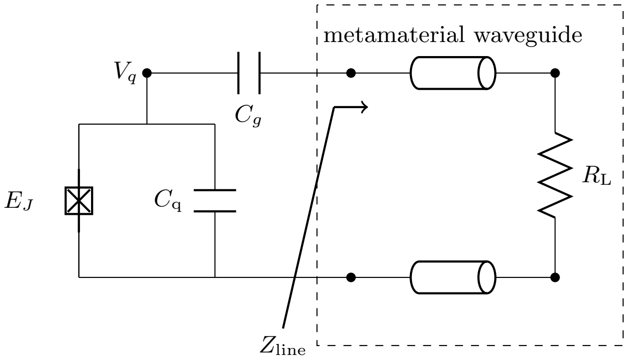

Supplementary Figure. 3. **Circuit diagram for a transmon qubit coupled to a metamaterial waveguide** The resistive termination is used to model radiation into the 50Ω coplanar waveguide.

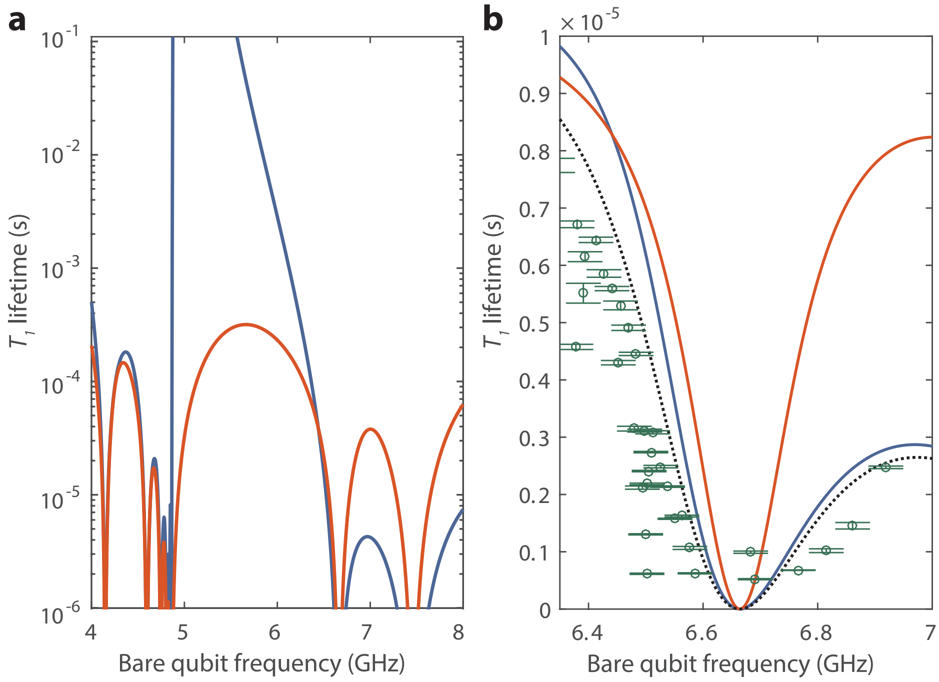

Supplementary Figure. 4. **Qubit lifetime as a function of resonance frequency.** **a**, Simulated qubit lifetime set by radiation into the output CPW port (blue), and structural loss in the waveguide (red). **b**, Comparison of the experimental results (open circles) with the simulated qubit lifetime (solid and dashed lines) near the first resonance dip in the upper transmission band. The lifetime set by radiation into the output port and structural loss in the waveguide are shown as blue and red solid lines, respectively. Both of these contributions have been adjusted to include a frequency independent intrinsic qubit life time of 10.86 µs. The black dashed line shows the theoretical qubit excited state lifetime including all contributions.

band	frequency (GHz)	$g/2\pi$ (MHz)	Q_{e} ($\times 10^3$)	Q_{i} ($\times 10^3$)
lower	4.2131	15.3	49.47	74.99
lower	4.6012	19.74	35.09	76.25
lower	4.7395	18.14	43.58	75
lower	4.8044	16.53	94.17	75.59
lower	4.8373	14.03	152.06	73.77
lower	4.856	9.73	455.8	76.47
lower	4.8654	4.48	2100	72
upper	6.6768	39.44	15.74	68.06
upper	7.309	58.06	12.02	70.44

Supplementary Table. I. **Measured resonance parameters for metamaterial waveguide.** The values are measured for the waveguide of Figs. 2-4 in main text. The resonances are measured in reflection from the input 50-Ω CPW port. The qubit-resonance coupling, g, is inferred from the anti-crossing observed as the qubit is tuned through each waveguide resonance.

SUPPLEMENTARY NOTE 1. BAND STRUCTURE ANALYSIS

A. Quantization of a periodic resonator-loaded waveguide

We consider the case of a waveguide that is periodically loaded with microwave resonators. Supplementary Figure 1 depicts a unit cell for this configuration. The Lagrangian for this system can be readily written as [1]

$$L = \sum_n \Bigg[\frac{1}{2}C_0[\dot{\Phi}^{\rm a}_n]^2 - \frac{[\Phi^{\rm a}_n - \Phi^{\rm a}_{n-1}]^2}{2L_0} + \frac{1}{2}C_{\rm r}[\dot{\Phi}^{\rm b}_n]^2 + \frac{1}{2}C_{\rm g}[\dot{\Phi}^{\rm a}_n - \dot{\Phi}^{\rm b}_n]^2 - \frac{[\Phi^{\rm b}_n]^2}{2L_{\rm r}}\Bigg]. \tag{1}$$

In order to find solutions in form of traveling waves, it is easier to work with the Fourier transform of node fluxes. We use the following convention for defining the (discrete) Fourier transformation

$$\Phi^{\rm a,b}_\kappa = \frac{1}{\sqrt{M}}\sum_{n=-N}^{N} e^{-i2\pi(\kappa/M)n}\Phi^{\rm a,b}_n, \tag{2}$$

where $M = 2N+1$ is the total number of periods in the waveguide. Using the Fourier relation we find the Lagrangian in k-space as

$$L = \sum_\kappa \Bigg[\frac{1}{2}(C_0 + C_{\rm g})|\dot{\Phi}^{\rm a}_\kappa|^2 - \left|1 - e^{-i2\pi(\kappa/M)}\right|^2 \frac{|\Phi^{\rm a}_\kappa|^2}{2L_0} \\ \frac{1}{2}(C_{\rm g} + C_{\rm r})|\dot{\Phi}^{\rm b}_\kappa|^2 - \frac{|\Phi^{\rm b}_\kappa|^2}{2L_{\rm r}} - C_{\rm g}\frac{\dot{\Phi}^{\rm b}_\kappa\dot{\Phi}^{\rm a}_{-\kappa} + \dot{\Phi}^{\rm b}_{-\kappa}\dot{\Phi}^{\rm a}_\kappa}{2}\Bigg]. \tag{3}$$

To proceed further, we need to find the canonical node charges which are defined as $Q^{\rm a,b}_\kappa = \frac{\partial L}{\partial \dot{\Phi}^{\rm a,b}_\kappa}$, and subsequently derive the Hamiltonian of the system by using a Legendre transformation. Doing so we find

$$H = \sum_\kappa \Bigg[\frac{Q^{\rm a}_\kappa Q^{\rm a}_{-\kappa}}{2C'_0} + \left|1 - e^{-i2\pi(\kappa/M)}\right|^2 \frac{\Phi^{\rm a}_\kappa\Phi^{\rm a}_{-\kappa}}{2L_0} + \\ \frac{Q^{\rm b}_\kappa Q^{\rm b}_{-\kappa}}{2C'_{\rm r}} + \frac{\Phi^{\rm b}_\kappa\Phi^{\rm b}_{-\kappa}}{2L_{\rm r}} + \frac{Q^{\rm a}_\kappa Q^{\rm b}_{-\kappa} + Q^{\rm a}_{-\kappa}Q^{\rm b}_\kappa}{2C'_{\rm g}}\Bigg]. \tag{4}$$

Here, we have defined the following quantities

$$C'_0 = \frac{C_{\rm g}C_{\rm r} + C_{\rm g}C_0 + C_0C_{\rm r}}{C_{\rm g} + C_{\rm r}}, \tag{5}$$

$$C'_{\rm r} = \frac{C_{\rm g}C_{\rm r} + C_{\rm g}C_0 + C_0C_{\rm r}}{C_{\rm g} + C_0}, \tag{6}$$

$$C'_{\rm g} = \frac{C_{\rm g}C_{\rm r} + C_{\rm g}C_0 + C_0C_{\rm r}}{C_{\rm g}}. \tag{7}$$

The canonical commutation relation $[\Phi^i_\kappa, Q^j_{-\kappa'}] = i\hbar\delta_{i,j}\delta_{\kappa,\kappa'}$ allows us to define the following annihilation operators as a function of charge and flux operators

$$\hat{a}_\kappa = \sqrt{\frac{C'_0\Omega_k}{2\hbar}}\left(\Phi^{\rm a}_\kappa + \frac{i}{C'_0\Omega_k}Q^{\rm a}_\kappa\right), \tag{8}$$

$$\hat{b}_\kappa = \sqrt{\frac{C'_{\rm r}\omega_0}{2\hbar}}\left(\Phi^{\rm b}_\kappa + \frac{i}{C'_{\rm r}\omega_0}Q^{\rm b}_\kappa\right). \tag{9}$$

Here, we have defined the resonance frequency for each mode as

$$\Omega_k = \sqrt{\frac{4\sin^2(kd/2)}{L_0C'_0}}, \tag{10}$$

$$\omega_0 = \frac{1}{\sqrt{L_{\rm r}C'_{\rm r}}}, \tag{11}$$

where $k = (2\pi\kappa)/(Md)$ is the wavenumber. It is evident that Ω_k has the expected dispersion relation of a discrete periodic transmission line and ω_0 is the resonance frequency of the loaded microwave resonators. Using the above definitions for $\hat{a}_\kappa, \hat{b}_\kappa$

$$\hat{H} = \frac{\hbar}{2}\sum_k \Big[\Omega_k\left(\hat{a}_k^\dagger\hat{a}_k + \hat{a}_{-k}\hat{a}_{-k}^\dagger\right) + \omega_0\left(\hat{b}_k^\dagger\hat{b}_k + \hat{b}_{-k}\hat{b}_{-k}^\dagger\right) \\ - g_k\left(\hat{b}_{-k} - \hat{b}_k^\dagger\right)\left(\hat{a}_k - \hat{a}_{-k}^\dagger\right) - g_k\left(\hat{a}_k^\dagger - \hat{a}_{-k}\right)\left(\hat{b}_{-k}^\dagger - \hat{b}_k\right)\Big], \tag{12}$$

along with the coupling coefficient

$$g_k = \frac{\sqrt{C_0' C_\mathrm{r}'}}{2C_\mathrm{g}'}\sqrt{\omega_0\Omega_k} = \frac{C_\mathrm{g}\sqrt{\omega_0\Omega_k}}{2\sqrt{(C_0 + C_\mathrm{g})(C_\mathrm{r} + C_\mathrm{g})}}. \tag{13}$$

An alternative structure for coupling microwave resonators is depicted in the bottom panel of Supplementary Figure 1. In this geometry, the coupling is controlled by the inductive element L_g. Repeating the analysis above for this case, we find

$$\Omega_k = \sqrt{\frac{4\sin^2(kd/2)}{C_0 L_0'}}, \tag{14}$$

$$\omega_0 = \frac{1}{\sqrt{C_\mathrm{r} L_\mathrm{r}'}}, \tag{15}$$

$$g_k = \frac{\sqrt{L_0' L_\mathrm{r}'}}{2L_\mathrm{g}'}\sqrt{\omega_0\Omega_k}. \tag{16}$$

We have defined the modified inductance values as

$$L_0' = \frac{L_\mathrm{g}L_\mathrm{r} + LL_\mathrm{g}L_0 + L_0L_\mathrm{r}}{L_\mathrm{g} + L_\mathrm{r}}, \tag{17}$$

$$L_\mathrm{r}' = \frac{L_\mathrm{g}L_\mathrm{r} + L_\mathrm{g}L_0 + L_0L_\mathrm{r}}{L_\mathrm{g} + L_0}, \tag{18}$$

$$L_\mathrm{g}' = \frac{L_\mathrm{g}L_\mathrm{r} + L_\mathrm{g}L_0 + L_0L_\mathrm{r}}{L_\mathrm{g}}. \tag{19}$$

B. Band structure calculation with RWA

Using the rotating wave approximation, the Hamiltonian in Eq. (12) can be simplified to

$$\hat{H} = \hbar\sum_k\left[\Omega_k\hat{a}_k^\dagger\hat{a}_k + \omega_0\hat{b}_k^\dagger\hat{b}_k + g_k\left(\hat{b}_k^\dagger\hat{a}_k + \hat{a}_k^\dagger\hat{b}_k\right)\right]. \tag{20}$$

Note that this approximation is applicable only when the coupling is sufficiently weak, $g_k \ll \min(\omega_0, \Omega_k)$, and the detuning is sufficiently small $|\omega_0 - \Omega_k| \ll (\omega_0 + \Omega_k)$. Assuming Ω_k and ω_0 are of the same order, this condition is satisfied when $C_\mathrm{g} \ll 2\sqrt{(C_0C_\mathrm{r})}$.

The simplified Hamiltonian can be written in the compact form

$$\hat{H} = \hbar\sum_k \mathbf{x}_k^\dagger\mathbf{H}_k\mathbf{x}_k, \tag{21}$$

where

$$\mathbf{H}_k = \begin{bmatrix}\Omega_k & g_k\\ g_k & \omega_0\end{bmatrix}, \mathbf{x}_k = \begin{bmatrix}\hat{a}_k\\ \hat{b}_k\end{bmatrix}. \tag{22}$$

We desire to transform the Hamiltonian to a diagonalized form

$$\tilde{\mathbf{H}}_k = \begin{bmatrix} \omega_{+,k} & 0 \\ 0 & \omega_{-,k} \end{bmatrix}. \tag{23}$$

It is straightforward to use the eigenvalue decomposition to find $\omega_{\pm,k}$ as

$$\omega_{\pm,k} = \frac{1}{2}\left[(\Omega_k + \omega_0) \pm \sqrt{(\Omega_k - \omega_0)^2 + 4g_k^2}\right], \tag{24}$$

along with the corresponding eigenstates $|\pm, k\rangle = \hat{\alpha}_{\pm,k}|0\rangle$, where

$$\hat{\alpha}_{\pm,k} = \frac{(\omega_{\pm,k} - \omega_0)}{\sqrt{(\omega_{\pm,k} - \omega_0)^2 + g_k^2}}\hat{a}_k + \frac{g_k}{\sqrt{(\omega_{\pm,k} - \omega_0)^2 + g_k^2}}\hat{b}_k. \tag{25}$$

C. Band structure calculation beyond RWA

The exact Hamiltonian in Eq. (12) can be written in the compact form

$$\hat{H} = \frac{\hbar}{2}\sum_k \mathbf{x}_k^\dagger \mathbf{H}_k \mathbf{x}_k, \tag{26}$$

where

$$\mathbf{H}_k = \begin{bmatrix} \Omega_k & 0 & g_k & -g_k \\ 0 & \Omega_k & -g_k & g_k \\ g_k & -g_k & \omega_0 & 0 \\ -g_k & g_k & 0 & \omega_0 \end{bmatrix}, \mathbf{x}_k = \begin{bmatrix} \hat{a}_k \\ \hat{a}_{-k}^\dagger \\ \hat{b}_k \\ \hat{b}_{-k}^\dagger \end{bmatrix}. \tag{27}$$

To find the eigenstates of the system, we can use a linear transform to map the state vector $\tilde{\mathbf{x}}_k = \mathbf{S}_k\mathbf{x}_k$ such that $\mathbf{x}_k^\dagger \mathbf{H}_k \mathbf{x}_k = \tilde{\mathbf{x}}_k^\dagger \tilde{\mathbf{H}}_k \tilde{\mathbf{x}}_k$ with the transformed diagonal Hamiltonian matrix

$$\tilde{\mathbf{H}}_k = \begin{bmatrix} \omega_{+,k} & 0 & 0 & 0 \\ 0 & \omega_{+,k} & 0 & 0 \\ 0 & 0 & \omega_{-,k} & 0 \\ 0 & 0 & 0 & \omega_{-,k}. \end{bmatrix} \tag{28}$$

In order to preserve the canonical commutation relations, the matrix $\mathbf{S}_k$ has to be symplectic, i.e. $\mathbf{J} = \mathbf{S}_k\mathbf{J}\mathbf{S}_k^\dagger$, with the matrix $\mathbf{J}$ defined as

$$\mathbf{J} = \begin{bmatrix} 1 & 0 & 0 & 0 \\ 0 & -1 & 0 & 0 \\ 0 & 0 & 1 & 0 \\ 0 & 0 & 0 & -1. \end{bmatrix} \tag{29}$$

A linear transformation (such as $\mathbf{S}_k$) that diagonalizes a set of quadratically coupled boson fields while preserving their canonical commutation relations is often referred to as a Bogoliubov-Valatin transformation. While it is generally difficult to find the transform matrix $\mathbf{S}_k$, it is easy to find the eigenvalues of the diagonalized Hamiltonian by exploiting some of the properties of $\mathbf{S}_k$. Note that since $\mathbf{J} = \mathbf{S}_k\mathbf{J}\mathbf{S}_k^\dagger$, the matrices $\mathbf{J}\tilde{\mathbf{H}}_k$ and $\mathbf{J}\mathbf{H}_k$ share the same set of eigenvalues. The eigenvalues of $\mathbf{J}\tilde{\mathbf{H}}_k$ are the two frequencies $\omega_{\pm,k}$, and thus we have

$$\omega_{\pm,k}^2 = \frac{1}{2}\left[(\Omega_k^2 + \omega_0^2) \pm \sqrt{(\Omega_k^2 - \omega_0^2)^2 + 16\omega_0\Omega_k g_k^2}\right]. \tag{30}$$

D. Circuit theory derivation of the band structure

Consider the pair of equations that describe the propagation of a monochromatic electromagnetic wave of the form $v(x,t) = V(x)e^{-ikx}e^{i\omega t}$ (along with the corresponding current relation) inside a transmission line

$$\frac{\mathrm{d}}{\mathrm{d}x}V(x) = -Z(\omega)I(x),$$
$$\frac{\mathrm{d}}{\mathrm{d}x}I(x) = -Y(\omega)V(x). \tag{31}$$

Here, $Z(\omega)$ and $Y(\omega)$ are frequency dependent impedance and admittance functions that model the linear response of the series and parallel portions of a transmission line with length d. It is straightforward to check that the solutions to these equation satisfy $k(\omega) = n\omega/c = \sqrt{-Z(\omega)Y(\omega)}/d$. For a loss-less waveguide and in the absence of dispersion we have $Z(\omega) = i\omega L_0$ and $Y(\omega) = i\omega C_0$, and thus we find the familiar dispersion relation $k(\omega) = \omega\sqrt{L_0 C_0}/d$. Nevertheless, the pair of equations above remain valid for arbitrary impedance and admittance functions $Z(\omega)$ and $Y(\omega)$, provided that the dimension of the model circuit remains much smaller than the wavelength under consideration. In this model, a real and negative quantity for the product ZY results in an imaginary wavenumber and subsequently creates a stop band in the dispersion relation. This situation can be achieved by periodically loading a transmission line with an array of resonators [2, 3]. Assuming a unit length of d we find

$$k^2 = \left(\frac{\omega}{c}\right)^2 n^2 \left[1 + \frac{2c\gamma_\mathrm{e}}{nd}\frac{1}{\omega_0^2 - \omega^2}\right]. \tag{32}$$

Here, ω_0 is the resonance frequency, and γ_e is the external coupling decay rate of an individual resonator in the array. For moderate values of gap-midgap ratio (Δ/ω_m), the frequency gap can be found as

$$\Delta = \frac{c}{nd}\left(\frac{\gamma_\mathrm{e}}{\omega_0}\right), \tag{33}$$

and $\omega_m = \omega_0 + \Delta/2$. We have defined the gap as the range of frequencies where the wavenumber is imaginary.

Although a microwave resonator can be realized by using a two-elements LC-circuit, the three-element circuits in Supplementary Figure 1 provide an additional degree of freedom which enables setting the coupling γ_e independent of the resonance frequency ω_0. Using circuit theory, it is straightforward to show

$$\omega_0 = \frac{1}{\sqrt{L_\mathrm{r}(C_\mathrm{r} + C_\mathrm{g})}}, \tag{34}$$
$$\gamma_\mathrm{e} = \frac{Z_0}{2L_\mathrm{r}}\left(\frac{C_\mathrm{g}}{C_\mathrm{r} + C_\mathrm{g}}\right)^2. \tag{35}$$

Here, Z_0 is the characteristic impedance of the unloaded waveguide. It is easy to check that for small values of $C_\mathrm{g}/C_\mathrm{r}$, the resonance frequency is only a weak function of C_g. As a result, it is possible to adjust the coupling rate γ_e by setting the capacitor C_g while keeping the resonance frequency almost constant. Supplementary Figure 1 also depicts an alternative strategy for coupling microwave resonators to the waveguide. In this design, the inductive element L_g is used to set the coupling in a "current divider" geometry. We provide experimental results for implementation of bandgap waveguide based on both designs in the next section.

While the "continuum" model described above provides a heuristic explanation for formation of bandgap in a waveguide loaded with resonators, its results remains valid as far as $k \ll 2\pi/d$. To avoid this approximation, we can use the transfer matrix method to find the exact dispersion relation for a system with discrete periodic symmetry [4]. In this case Eq. (32) is modified to

$$\cos(kd) = 1 - \left(\frac{\omega}{c}\right)^2 \frac{n^2 d^2}{2} - \frac{nd\gamma_\mathrm{e}}{c}\frac{\omega^2}{\omega_0^2 - \omega^2}. \tag{36}$$

Note that this relation still requires d to be much smaller than the wavelength of the unloaded waveguide $\lambda = 2\pi c/(n\omega)$.

E. Dispersion and group index near the band-edges

Equation (30) can be reversed to find the wavenumber k as a function of frequency. Assuming, a linear dispersion relation of the form $k = n\Omega_k/c$ for the bare waveguide we find

$$k = \frac{n\omega}{c}\sqrt{\frac{\omega^2-\omega_{c+}^2}{\omega^2-\omega_{c-}^2}}. \tag{37}$$

Here, $\omega_{c+} = \omega_0$ and $\omega_{c-} = \omega_0\sqrt{1-4g_k^2/(\Omega_k\omega_0)}$ are the upper and lower cut-off frequencies, respectively. The quantity $g_k^2/(\Omega_k\omega_0)$ is a unit-less parameter quantifying the size of the bandgap and is independent of the wavenumber k.

The dispersion relation can be written in simpler forms by expanding the wavenumber in the vicinity of the two band-edges

$$k = \begin{cases} \frac{n\omega_{c-}}{c}\sqrt{\frac{\Delta}{-\delta_-}} & \text{for } \omega \approx \omega_{c-}, \\ \frac{n\omega_{c+}}{c}\sqrt{\frac{\delta_+}{\Delta}} & \text{for } \omega \approx \omega_{c+}. \end{cases} \tag{38}$$

Here, $\Delta = \omega_{c+} - \omega_{c-}$ is the frequency span of the bandgap and $\delta_\pm = \omega - \omega_{c\pm}$ are the detunings from the band-edges.

The form of the dispersion relation Eq. (30) suggests that the maxima of the group index happens near the band-edges. Having the wavenumber, we can readily evaluate the group velocity $v_g = \partial\omega/\partial k$ and find the group index $n_g = c/v_g$ as

$$n_g = \begin{cases} \frac{n\omega_{c-}\sqrt{\Delta}}{\sqrt{-4(\delta_- - i\gamma_i)^3}} & \text{for } \omega \approx \omega_{c-}, \\ \frac{n\omega_{c+}}{\sqrt{4\Delta(\delta_+ - i\gamma_i)}} & \text{for } \omega \approx \omega_{c+}. \end{cases} \tag{39}$$

Note that we have replaced $\delta_\pm$ with $\delta_\pm - i\gamma_i$ to account for finite internal quality factor of the resonators in the structure.

F. Coupling a Josephson junction qubit to a metamaterial waveguide

We consider the coupling of a Josephson junction qubit to the metamaterial waveguide. Assuming rotating wave approximation (valid for weak coupling $f_k \ll \omega_k, \omega_\mathrm{q}$), the Hamiltonian of this system can be written as

$$\hat{H} = \hbar\sum_k\left[\omega_k\hat{a}_k^\dagger\hat{a}_k + \frac{\omega_\mathrm{q}}{2}\hat{\sigma}_z + f_k\left(\hat{a}_k^\dagger\hat{\sigma}^- + \hat{a}_k\hat{\sigma}^+\right).\right] \tag{40}$$

Here f_k is the coupling factor of the qubit to the waveguide photons, and $\omega_k = \omega_{\pm,k}$, where the plus or minus sign is chosen such that the qubit frequency ω_q lies within the band. Without loss of generality, we assume f_k to be a real number. The Heisenberg equations of motions for the qubit and the photon operators can be written as

$$\frac{\partial}{\partial t}\hat{a}_k = -i\omega_k\hat{a}_k - if_k\hat{\sigma}^- \tag{41}$$

$$\frac{\partial}{\partial t}\hat{\sigma}^- = -i\omega_\mathrm{q}\hat{\sigma}^- - i\sum_k f_k\hat{a}_k \tag{42}$$

The equation for $\hat{a}_k$ can be formally integrated and substituted in the equation for $\hat{\sigma}^-$ to find

$$\frac{\partial}{\partial t}\hat{\sigma}^- = -i\omega_\mathrm{q}\hat{\sigma}^- - i\sum_k f_k e^{-i\omega_k(t-t_0)}\hat{a}_k(t_0) - \sum_k f_k^2\int_{t_0}^t e^{-i(\omega_k)(t-\tau)}\hat{\sigma}^-(\tau)\mathrm{d}\tau. \tag{43}$$

We now use the Markov approximation to write $\hat{\sigma}^-(\tau) \approx \hat{\sigma}^-(t)e^{-i(\omega_\mathrm{q})(\tau-t)}$, and thus

$$\frac{\partial}{\partial t}\hat{\sigma}^- = -i\omega_\mathrm{q}\hat{\sigma}^- - i\sum_k f_k e^{-i\omega_k(t-t_0)}\hat{a}_k(t_0) - \sum_k f_k^2\left(\int_{t_0}^t e^{-i(\omega_k-\omega_\mathrm{q})(t-\tau)}\mathrm{d}\tau\right)\hat{\sigma}^-(t). \tag{44}$$

Considering the generic equation of motion for a linearly decaying qubit, $(\partial/\partial t)\hat{\sigma}^- = -i\omega_\mathrm{q}\hat{\sigma}^- - (\gamma/2)\hat{\sigma}^-$, we can identify real part of the last term in the equation above as the decay rate due to radiation of the qubit into the waveguide. We can extend the integral's bound to approximately evaluate this term as

$$\begin{aligned}
\gamma &= 2\,\mathrm{Re}\left[\sum_k f_k^2 \int_{t_0}^{t} e^{-i(\omega_k-\omega_\mathrm{q})(t-\tau)}\mathrm{d}\tau\right] \\
&\approx 2\,\mathrm{Re}\left[\sum_k f_k^2 \int_{t_0}^{\infty} e^{-i(\omega_k-\omega_\mathrm{q})(t-\tau)}\mathrm{d}\tau\right] \\
&= 2\pi \sum_k f_k^2 \delta(\omega_k - \omega_\mathrm{q}).
\end{aligned} \tag{45}$$

Assuming the coupling rate f_k is a smooth function of the k-vector, we can evaluate this some in the continuum limit as

$$\gamma = 2\pi \sum_k f_k^2 \delta(\omega_k - \omega_\mathrm{q}) \tag{46}$$

$$\approx Md \int \mathrm{d}k f_k^2 \delta(\omega_k - \omega_\mathrm{q}) \tag{47}$$

$$= L \int \mathrm{d}\omega \left(\frac{\partial k}{\partial \omega}\right) f_k^2 \delta(\omega_k - \omega_\mathrm{q}) \tag{48}$$

$$= \frac{L}{c} f(\omega_\mathrm{q})^2 n_\mathrm{g}(\omega_\mathrm{q}). \tag{49}$$

It is evident that reducing the group velocity increases the radiation decay rate of the qubit.A similar analysis can be applied to find the decay rate of a linear cavity with resonance frequency of ω_0 (i.e. a harmonic oscillator) that has been coupled to the waveguide with coupling constant $g(\omega)$. In this case we find

$$\begin{aligned}
\gamma &= \frac{L}{c} g(\omega_0)^2 n_\mathrm{g}(\omega_0), \\
Q_\mathrm{e} &= \omega_0/\gamma = \frac{\omega_0 c}{L} \frac{1}{g(\omega_0)^2 n_\mathrm{g}(\omega_0)}.
\end{aligned} \tag{50}$$

SUPPLEMENTARY NOTE 2. CHARACTERIZATION AND MODELING OF THE METAMATERIAL WAVEGUIDE

Several competing effects in the design of the metamaterial waveguide influence its utility within a waveguide QED setting. We desire a compact waveguide unit cell to reduce the required real estate in a chip-scale platform. This should be combined with a large bandgap to provide more spectral bandwidth and tighter localization of photon bound states. We also require efficient qubit-waveguide coupling. These attributes allow for denser integration of qubits both in space and frequency, enabling larger-scale and more complex quantum circuits. They can be obtained in a single metamaterial design provided both the resonator elements and the waveguide sections are of high impedance, and that the waveguide section has large inductance. The logic for this is as follows. The bandgap of the metamaterial waveguide scales roughly with the product of the coupling capacitance, the zero-point voltage of the resonator, and the zero-point voltage of the waveguide section, $\Delta \propto C_k V_{\mathrm{zpf}}^{\mathrm{CPW}} V_{\mathrm{zpf}}^{\mathrm{res}}$. The zero-point voltage fluctuations scale with the impedance. Additionally, a large bandgap requires that the inductance of the waveguide section be large so that the resonant frequency of the bare waveguide section at the X-point ($kd = \pi/a$) is not too far detuned from that of the bare resonators, $\Omega_{k=\pi/da} = (L_0 C_0')^{-1/2} \sim \omega_{\mathrm{r}}$.

We obtain a large resonator impedance by using spiral inductors made from narrow cross-section wires of long coil length, with the impedance of the resonator roughly scaling as the inverse of the square root of the coil width (w), $Z_{\mathrm{res}} \sim 1/\sqrt{w}$. The impedance of the CPW line can be set by adjusting the ratio of the center conductor width to the physical gap between center conductor and the ground plane (smaller ratio yields higher impedance). In order to also realize a large inductance at the same time, without dramatically increasing the length of the waveguide section, we meander the center conductor of the waveguide section to give it more effective path length and larger inductance. In the devices presented in this work the period of the metamaterial waveguide is 350 µm, with the length of the waveguide section corresponding to 210 µm of this length. The wire width of the CPW center conductor (resonator coil) was chosen conservatively to be 5 µm (1 µm) to limit the potential disorder arising from fluctuations in the kinetic inductance due to wire width inhomogeneity.

Estimates of the resulting lumped element parameters obtained from fits to the measured transmission data of the fabricated metamaterial waveguide in Supplementary Figure 1 of the main text are: C_r = 345 fF, L_r = 1.43 nH, C_{g} = 389 fF, C_0 = 50.5 fF, L_0 = 0.7885 nH. These values are very close to the design values. From th measured widths of the waveguide resonances in the transmission bands, we find a good to fit to the loss in the waveguide by assuming a resistance $R_r = 8\times10^{-4}$ Ω in series with L_r and a resistance $R_0 = 4\times10^{-4}$ Ω in series with L_0. For the metamaterial waveguide coupled to the qubit of Figs. 2-4 in the main text we used a slightly different design, with estimated lumped-element parameters equal to: C_r = 240 fF, L_r = 2.10 nH, $R_r = 1.1 \times 10^{-3}$ Ω, C_{g} = 252 fF, C_0 = 52.0 fF, L_0 = 1.19 nH, $R_0 = 6\times10^{-4}$ Ω. Here, the fit parameters were inferred from the frequencies and linewidths of the lower and upper band resonances (within the 4-8 GHz circulator bandwidth of our set-up), measured in reflection from the input 50-Ω CPW port. The read-out of the qubit state has been performed using one of the two upper band modes (f = 6.67 GHz, and f = 7.3 GHz), depending on the frequency of the qubit at each flux bias point. The measured resonance parameters, along with their coupling (g) to the qubit, are tabulated in Tab. I. The qubit-to-waveguide coupling was designed and simulated to be given by a coupling capacitance of C_g = 4.8 fF. In all our circuit model fits this coupling capacitance was fixed at the design value, and not needed as a fitting parameter.

SUPPLEMENTARY NOTE 3. CHARACTERIZATION OF LUMPED-ELEMENT MICROWAVE RESONATORS

We have achieved a characteristic size of $\lambda_0/150$ (130 µm by 76 µm for $\omega_0/2\pi \approx 6$ GHz) and $\lambda_0/76$ (155 µm by 92 µm for $\omega_0/2\pi \approx 10$ GHz), using a wire width of 500 nm and 1 µm, respectively.

Supplementary Figure 2 shows the typical amplitude and phase of measured for a waveguide coupled to a pair of identical resonators. Microwave spectroscopy of the fabricated resonators is performed in a dilution refrigerator cooled-down to a temperature of $T_f \approx 7$ mK. The input microwave is launched onto the chip via a 50-Ω CPW. The output microwave signal is subsequently amplified and analyzed using a network analyzer (for more details regarding the measurement setup, refer to Ref. [5]). We have extracted the intrinsic and extrinsic decay rates of the cavity by fitting the transmission data to a Fano line shape of the form

$$S_{21}(\omega) = 1 - \frac{\gamma_\mathrm{e} e^{i\phi_0}}{\gamma_\mathrm{i} + \gamma_\mathrm{e} + 2i(\omega - \omega_0)}. \tag{51}$$

Here γ_e and γ_i are the extrinsic and intrinsic decay rates of the resonator, respectively. The phase ϕ_0 is a parameter that sets the asymmetry of the Fano line shape [6]. The data demonstrates that it is possible to adjust the external coupling to the resonator in a wide range without much degradation in the internal quality factor (it is straightforward to convert the extrinsic quality factor Q_e to the coupling constants g_k used in our theoretical analysis above). We have compared the measured resonance frequency with the resonance frequency found from numerical simulations in Supplementary Figure 2d. We find that the measured resonance frequencies are in agreement with the simulated values, with a multiplicative scaling factor of 0.85. Using this scale factor, we have measured a random variation 0.3% in the resonance frequency. It has been previously suggested that the shift in the resonance frequency and its statistical variation can be attributed to the kinetic inductance of the free charge carriers in the superconductor, and the variations can be mitigated by increasing the wire width [7].

SUPPLEMENTARY NOTE 4. DISORDER AND ANDERSON LOCALIZATION

Propagation of electron waves in a one dimensional quasi-periodic potential is described by

$$\left[-\frac{\partial^2}{\partial x^2}+\sum_n (U+U_n)\delta(x-an)\right]\psi_q(x)=q^2\psi_q(x). \tag{52}$$

Here, q is the quasi momentum and U_n is the random variable that models compositional disorder at position $x=na$. Disorder leads to localization of waves with a characteristic length defined as

$$\ell^{-1}=\lim_{N\to\infty}\left\langle \frac{1}{N}\sum_{n=0}^{N-1}\ln\left|\frac{\psi_{n+1}}{\psi_n}\right|\right\rangle. \tag{53}$$

Here, the brackets represent averaging over different realization of the disorder, whereas the summation accounts for spatial/temporal averaging for traveling waves. For this model, previous authors have found the localization length to be [8–10]

$$\frac{\ell}{d}=\frac{2\Gamma(1/6)}{6^{1/3}\sqrt{\pi}}\sigma^{-2/3}\approx 3.45\sigma^{-2/3}. \tag{54}$$

In this model $\sigma^2=\langle U_n^2\rangle\sin^2(q_0a)/q_0^2$ is a parameter that quantifies the strength of disorder, and q_0 is the value of quasi-momentum at the band-edge.

Now, we consider the propagation of current waves in a one dimensional waveguide that has been periodically loaded with resonators (a similar analysis can be applied to the voltage waves for the case of inductively coupled resonators). Starting from Eq. (32), it is straightforward to find

$$\frac{\partial^2 I(x)}{\partial x^2}+I(x)\Big(\frac{\omega}{c}\Big)^2 n^2\left[1+\sum_n\frac{d\Delta\delta(x-an)}{\omega_{0,n}-\omega+i\gamma_\mathrm{i}}\right]=0. \tag{55}$$

By comparing this equation with the Schrodinger equation for the Kronig-Penny model Eq. (52) we find

$$\begin{aligned} q^2 &\to \Big(\frac{\omega}{c}\Big)^2 n^2 \\ U+U_n &\to -\Big(\frac{\omega}{c}\Big)^2 n^2\left[\frac{d\Delta}{\omega_{0,n}-\omega+i\gamma_\mathrm{i}}\right]. \end{aligned} \tag{56}$$

For small variation in resonance frequencies, $\delta\omega_0$, we can expand the resonance potential term to find

$$U_n=-\Big(\frac{\omega_0}{c}\Big)^2 n^2\frac{\partial}{\partial\omega_{0,n}}\left(\frac{d\Delta}{\omega_{0,n}-\omega+i\gamma_\mathrm{i}}\right)\delta\omega_0 \tag{57}$$

By evaluating the expression for U_n and substituting it in the relation above for σ^2, we find

$$\begin{aligned} \sigma^2_\mathrm{low} &= \left(\frac{\gamma_\mathrm{e}}{\gamma_\mathrm{i}}\right)^4\left(\frac{\delta\omega_0}{\Delta}\right)^2, \\ \sigma^2_\mathrm{high} &= \left(\frac{\gamma_\mathrm{e}}{\Delta}\right)^4\left(\frac{\delta\omega_0}{\Delta}\right)^2. \end{aligned} \tag{58}$$

The analysis above gives us ℓ_dis. In addition to disorder, absorption loss in the metamaterial waveguide components (specifically the resonators) leads to an exponential extinction of the wave's amplitude. An effective localization length incorporating absorption loss, ℓ_loss, can be found by solving for the complex band structure and setting $\ell_\mathrm{loss}=1/\mathrm{Im}(k)$. For propagation of a classical wave through the waveguide both loss and disorder contribute to exponential extinction of the wave with a total localization length of

$$\frac{1}{\ell_\mathrm{total}}=\frac{1}{\ell_\mathrm{dis}}+\frac{1}{\ell_\mathrm{loss}}. \tag{59}$$

Two important points should be made here. First, ℓ_{loss} as defined is purely an absorption loss effect only outside any photonic bandgap region. Inside a photonic bandgap the periodic loading of the waveguide gives rise to an imaginary k-vector as well. As such, ℓ_{loss} inside the gap will contain both periodic loading effects and absorption loss effects. Second, the exponential localization of the photonic wavefunction caused by the periodic loading of the waveguide and the localization caused by structural disorder are coherent (unitary) effects. On the contrary, the exponential attenuation of a traveling wavepacket due to the loss in the resonators is a dissipative effect. When considering photon-mediated interactions between qubits, these two effects for the most part need to be addressed separately. In this context, the value of ℓ_{total} as a single parameter is limited to primarily estimating the spatial extent over which strong coherent interactions can be obtained.

SUPPLEMENTARY NOTE 5. QUBIT FREQUENCY SHIFT AND LIFETIME

G. Circuit theory modeling

The qubit frequency shift can be derived from circuit theory by modeling the qubit as a linear resonator. Consider the circuit diagram in Supplementary Figure 3. The load impedance seen from the qubit port can be written as

$$Z_{\mathrm{L}}(\omega) = \frac{1}{i\omega C_{\mathrm{g}}} + Z_{\mathrm{line}}(\omega), \tag{60}$$

and

$$Y_{\mathrm{L}}(\omega) = \frac{i\omega C_{\mathrm{g}}}{1 + Z_{\mathrm{line}}(\omega) i\omega C_{\mathrm{g}}}. \tag{61}$$

For weak coupling, the decay rate can be found using the real part of the load impedance as

$$\kappa \simeq \omega_{\mathrm{q}}^2 L_{\mathrm{J}} \,\mathrm{Re}\,[Y_L(\omega_{\mathrm{q}})]. \tag{62}$$

Here, ω_{q} is the resonance frequency of the qubit. Similarly, the shift in qubit frequency is found as

$$\Delta\omega_{\mathrm{q}} \simeq -\frac{\omega_{\mathrm{q}}^2 L_{\mathrm{J}}}{2} \,\mathrm{Im}\,[Y_L(\omega_{\mathrm{q}})]\,. \tag{63}$$

For a transmon qubit, we have the following relation that approximate its behavior in the linearized regime

$$L_{\mathrm{J}} = \frac{\left(\frac{\Phi_0}{2\pi}\right)^2}{E_{\mathrm{J}}}, \tag{64}$$

$$\omega_{\mathrm{q}} = \frac{1}{\sqrt{L_{\mathrm{J}} C_{\mathrm{q}}}}. \tag{65}$$

We first use the simplified continuum model to find the input impedance Z_{line}

$$Z_{\mathrm{line}}(\omega) = Z_{\mathrm{B}}(\omega) \frac{R_{\mathrm{L}} + Z_{\mathrm{B}}(\omega) \tanh\left[\mathrm{Im}\,(k)x\right]}{Z_{\mathrm{B}}(\omega) + R_{\mathrm{L}} \tanh\left[\mathrm{Im}\,(k)x\right]}. \tag{66}$$

Here, $\mathrm{Im}\,(k)(\omega)$ is the attenuation constant (we are assuming $\mathrm{Re}\,(k)(\omega) = 0$, i.e. valid when the value of ω is within the bandgap), $Z_{\mathrm{B}}(\omega)$ is the Bloch impedance of the periodic structure, and x is the length of the waveguide. Assuming $\mathrm{Im}\,(k)x \gg 1$, this expression can be simplified as

$$\begin{aligned} Z_{\mathrm{line}}(\omega) \approx & Z_{\mathrm{B}}(\omega) + \frac{4R_{\mathrm{L}} |Z_{\mathrm{B}}(\omega)|^2}{{R_{\mathrm{L}}}^2 + |Z_{\mathrm{B}}(\omega)|^2} e^{-2\,\mathrm{Im}\,(k)x} \\ \approx & Z_{\mathrm{B}}(\omega) + 4R_{\mathrm{L}} e^{-2\,\mathrm{Im}\,(k)x}. \end{aligned} \tag{67}$$

Note that we have assumed $R_{\mathrm{L}} \ll |Z_{\mathrm{B}}(\omega)|$ to make the last approximation. For weak coupling, the qubit coupling capacitance, C_{g}, should be chosen such that the (magnitude of) impedance $Z_{\mathrm{g}} = 1/(i\omega C_{\mathrm{g}})$ is much larger than $|Z_{\mathrm{line}}|$. In this situation, we use Eq. (63) and Eq. (67) to find

$$\begin{aligned} \frac{\Delta\omega_{\mathrm{q}}}{\omega_{\mathrm{q}}} &= -\frac{1}{2}(L_{\mathrm{J}}\omega_{\mathrm{q}})(C_{\mathrm{g}}\omega_{\mathrm{q}}) - \frac{1}{2}(L_{\mathrm{J}}\omega_{\mathrm{q}})(C_{\mathrm{g}}\omega_{\mathrm{q}})^2 \mathrm{Im}[Z_{\mathrm{B}}(\omega_{\mathrm{q}})] \\ &= -\frac{C_{\mathrm{g}}}{2C_{\mathrm{q}}} - \frac{C_{\mathrm{g}}}{2C_{\mathrm{q}}} \mathrm{Im}[Z_{\mathrm{B}}(\omega_{\mathrm{q}})] C_{\mathrm{g}}\omega_{\mathrm{q}}. \end{aligned} \tag{68}$$

Note that the first term in the frequency shift is merely caused by addition of the coupling capacitor to the overall qubit capacitance.

We find the qubit's decay rate caused by radiation into the output port by substituting Eq. (67) in Eq. (62)

$$\kappa_{\mathrm{rad}} = \frac{4\omega_{\mathrm{q}}^2 {C_{\mathrm{g}}}^2}{C_{\mathrm{q}}} R_{\mathrm{L}} e^{-2\,\mathrm{Im}\,(k)(\omega)x}. \tag{69}$$

Subsequently, the lifetime of the qubit can be written as

$$T_{\mathrm{rad}} = \frac{C_{\mathrm{q}}}{4\omega_{\mathrm{q}}^2 {C_{\mathrm{g}}}^2 R_{\mathrm{L}}} e^{2x/\ell(\omega_{\mathrm{q}})}, \tag{70}$$

where $\ell = 1/\,\mathrm{Im}\,(k)$ is the localization length in the bandgap. We note that the analysis from circuit theory is only valid for weak qubit-waveguide coupling rates, where the Markov approximation can be applied. In the strong coupling regime, the qubit frequency and lifetime can be found by numerically finding the zeros of the circuit's admittance function $Y = Y_{\mathrm{L}} + Y_{\mathrm{q}}$, where $Y_{\mathrm{q}} = i\omega_{\mathrm{q}} C_{\mathrm{q}} + 1/(i\omega_{\mathrm{q}} L_{\mathrm{J}})$.

H. Effect of structural loss in the waveguide on the qubit lifetime

Equation (69) gives the decay rate of qubit's excited state caused by radiation into the output CPW port. In addition to this radiative component, the loss in the waveguide also contributes to the decay rate of the qubit excited state. The effect of loss in the waveguide can be modeled as the (incoherent) sum of contributions to the decay rate from the individual resonances in the transmission bands of the waveguide:

$$\kappa_{\mathrm{loss}} = \sum_m \frac{{g_m}^2 \kappa_{i,m}}{2{g_m}^2 + \mid \kappa_{i,m} - i(\omega_{\mathrm{q}} - \omega_{0,m}) \mid^2}. \tag{71}$$

Here, m denotes the index of each waveguide resonance, g_m is the coupling rate of the qubit to the waveguide resonance, and $\kappa_{i,m}$ is the intrinsic decay rate for each waveguide resonance. The parameters ω_{q} and $\omega_{0,m}$ denote the fundamental transition frequency of the qubit and the resonance frequency of the waveguide mode, respectively. For a finite waveguide made from 9 unit cells we expect a total of 18 resonances, with half of them distributed in the lower frequency transmission band and half of them distributed in the upper transmission band. Table I presents the measured $\omega_{0,m}$, $Q_{i,m} = \omega_{0,m}/\kappa_{i,m}$, and g_m parameters for the 9 resonances closest to the waveguide bandgap that are observable in the frequency band of the circulators used in our experiment. The total lifetime of the qubit excited state can be determined from $T_1 = 1/(\kappa_{\mathrm{rad}} + \kappa_{\mathrm{loss}} + \kappa_{\mathrm{i}})$, where κ_i represents a third decay channel for the qubit corresponding to coupling to all other degrees of freedom (two-level systems, etc.). From a fit to the measured qubit T_1 data deep in the bandgap we find an intrinsic lifetime of $\kappa_{\mathrm{i}}^{-1} = 10.86\,\mu\mathrm{s}$.

To identify regions of frequency space where the qubit lifetime is limited by output port radiation, and therefore may tell us about the finite extent of the photon wavepacket coupled to the qubit, we plot in Supplementary Figure 4a the estimated loss and output radiation contributions to the qubit lifetime as a function of frequency in and around the waveguide bandgap for our experiment. It is evident the internal waveguide loss contribution is the dominant factor deep in the band gap and near the lower transmission band-edge, where the localization length of the photon wavepacket coupled to the qubit is much smaller than the finite length of the waveguide. On the contrary, the radiation into the output CPW port is the dominant factor near the upper band-edge frequency in the band gap and inside the upper transmission band, where the localization length becomes comparable and larger than the finite length of the waveguide. A zoomed-in plot around the upper band-edge frequency of the measured qubit excited state lifetime along with the different estimated components of the qubit decay are shown in Supplementary Figure 4b. The asymmetric profile of the measured lifetime near the first resonance in the upper transmission band is a clear sign of the radiation into the output port, where the shorter lifetime for frequencies above the resonance frequency can be attributed to coherent (constructive) interference from multiple waveguide resonances. These subtle features help differentiate single mode and incoherent multi-mode cavity-QED effects, from true waveguide-QED effects in which multi-mode interference leads to radiative dynamics governed by a localized photon wavepacket.

SUPPLEMENTARY NOTE 6. GROUP DELAY AND THE QUBIT LIFETIME PROFILE

Equation (70) demonstrates the relation between the qubit lifetime and the localization length. Moving the qubit frequency beyond the gap, results in a drastic increase in the localization length and subsequently reduces the qubit lifetime. The normalized slope of the lifetime profile in the vicinity of the band-edge can be written as

$$\left|\frac{1}{T_{\mathrm{rad}}}\frac{\partial T_{\mathrm{rad}}}{\partial \omega}\right| = \left|x\frac{\partial\,\mathrm{Im}\,(k)}{\partial \omega}\right| = \left|x\,\mathrm{Im}\,(n_{\mathrm{g}})/c\right|. \tag{72}$$

We now evaluate Eq. (39) to find the group index at the upper and lower band-edges $\delta_{\pm} = 0$

$$|\,\mathrm{Re}(n_{\mathrm{g}})| = |\,\mathrm{Im}(n_{\mathrm{g}})| = \begin{cases} n\omega_{\mathrm{c}-}\sqrt{\frac{\Delta}{8{\gamma_{\mathrm{i}}}^{3}}} & \text{for } \omega = \omega_{\mathrm{c}-}, \\ n\omega_{\mathrm{c}+}\frac{1}{\sqrt{8\Delta\gamma_{\mathrm{i}}}} & \text{for } \omega = \omega_{\mathrm{c}+}. \end{cases} \tag{73}$$

Consequently, we can write the normalized slope of the lifetime profile at the band-edge as

$$\begin{aligned}\left(\frac{1}{T_{\mathrm{rad}}}\left|\frac{\partial T_{\mathrm{rad}}}{\partial \omega}\right|\right)\bigg|_{\omega=\omega_{c\pm}} &= \left|x\,\mathrm{Im}\left[n_{\mathrm{g}}(\omega_{c\pm})\right]/c\right| \\ &= \left|x\,\mathrm{Re}\left[n_{\mathrm{g}}(\omega_{c\pm})\right]/c\right| \\ &= \tau_{\mathrm{delay}}.\end{aligned} \tag{74}$$

This result has a simple description: the normalized slope of the lifetime profile at the band-edge is equal to the (maximum) group delay.

SUPPLEMENTARY NOTE 7. SCALING THE WAVEGUIDE LENGTH

Scaling the length of the waveguide to the extreme limits requires dealing with a number of technical challenges. Below, we outline a number of these challenges and possible strategies for addressing them. A systematic study of these challenges and efficient strategies for overcoming them will be the subject of a future study.

Resonator size: The size of lumped-element resonators is ultimately limited by the fabrication considerations for thin-film aluminum nano-wires. A pitch size of 60 nm can be achieved in these structures by using electron beam lithography for patterning the wires [11]. Assuming a quarter-wave resonator geometry, and a wire-to-airgap ratio of unity, we find the characteristic size for the resonator as $d = \sqrt{(60\text{nm} \times \lambda/4)}$. Using $\lambda = 2$ cm for a 6 GHz resonator (on Si substrate), we have $d = \lambda/1100$. An alternative strategy for miniaturizing the resonators is to use kinetic inductance of disordered superconductors [12].

We emphasize that using smaller resonators requires a careful study of disorder in the resonance frequency, and possible strategies for reducing it.

Bandgap size: The lumped-element nature of the components in our device allows for achieving a larger bandgap-to-midgap ratio by simply increasing the coupling capacitor, C_g, and reducing the internal capacitance, C_r. Ultimately, however, the gap size will be limited by the minimum value of C_r which is itself set by the parasitic capacitance of the inductor in the resonator (L_r). Considering the numerical values of these quantities listed in the Supplementary Note 2, we anticipate that a two-fold increase in the bandgap-to-midgap is feasible.

Parasitic modes: The placement of the qubit with respect to the metamaterial waveguide in our device relies on the symmetry of the waveguide modes to eliminate coupling to the parasitic modes of the structure. Alternatively, Aluminum air bridges can be implemented in the coplanar waveguide (CPW) sections of our device in order to suppress the slot-line modes of the waveguide. Suppressing the slot-line modes allows for realizing a more flexible geometry where multiple qubits can be capacitvely coupled to resonators along the waveguide.

Making turns with the metamaterial waveguide: Effective use of chip's area requires the ability to make $90°/180°$ turns along the path for long waveguides. In our device, turns can be implemented by modifying the CPW sections between the cavities. To this end, the meandered coplanar waveguide between the cavities can be unwrapped to realize turns with a radius of curvature 50 um-200 um. The resulting asymmetry and local frequency shift caused by the bend can be compensated by numerical modeling of these effects and making proper adjustments to the neighboring resonators.

Supplementary References

[1] M. H. Devoret, “Quantum fluctuations in electrical circuits,” *Les Houches Lectures*, 1995.
[2] B. M. Karyamapudi and J.-S. Hong, “Coplanar waveguide periodic structures with resonant elements and their application in microwave filters,” *IEEE MTT-S International Microwave Symposium - IMS 2003*, vol. 3, pp. 1619–1622, 2003.
[3] K. O’Brien, C. Macklin, I. Siddiqi, and X. Zhang, “Resonant Phase Matching of Josephson Junction Traveling Wave Parametric Amplifiers,” *Phys. Rev. Lett.*, vol. 113, no. 15, p. 157001, Oct. 2014.
[4] D. M. Pozar, *Microwave Engineering, 4th.* Edition-John Wiley, 1998.
[5] P. B. Dieterle, M. Kalaee, J. M. Fink, and O. Painter, “Superconducting Cavity Electromechanics on a Silicon-on-Insulator Platform,” *Phys. Rev. Applied*, vol. 6, no. 1, p. 014013, Jul. 2016.
[6] J. Gao, “The physics of superconducting microwave resonators,” Ph.D. dissertation, California Institute of Technology, 2008.
[7] D. L. Underwood, W. E. Shanks, J. Koch, and A. A. Houck, “Low-disorder microwave cavity lattices for quantum simulation with photons,” *Phys. Rev. A*, vol. 86, no. 2, p. 023837, Aug. 2012.
[8] J. C. Hernández-Herrejón, F. M. Izrailev, and L. Tessieri, “Anomalous localization in the aperiodic Kronig–Penney model,” *Journal of Physics A: Mathematical and Theoretical*, vol. 43, no. 42, p. 425004, Oct. 2010.
[9] V. Dossetti-Romero, F. M. Izrailev, and A. A. Krokhin, “Transport properties of 1D tight-binding disordered models: the Hamiltonian map approach,” *Physica E: Low-dimensional Systems and Nanostructures*, vol. 25, no. 1, pp. 13–22, Oct. 2004.
[10] F. M. Izrailev, T. Kottos, and G. P. Tsironis, “Hamiltonian map approach to resonant states in paired correlated binary alloys,” *Phys. Rev. B*, vol. 52, no. 5, pp. 3274–3279, Aug. 1995.
[11] N. A. Melosh, A. Boukai, F. Diana, B. Gerardot, A. Badolato, P. M. Petroff, and J. R. Heath, “Ultrahigh-density nanowire lattices and circuits,” *Science (New York, N.Y.)*, vol. 300, no. 5616, pp. 112–115, 2003.
[12] N. Samkharadze, A. Bruno, P. Scarlino, G. Zheng, D. P. DiVincenzo, L. DiCarlo, and L. M. K. Vandersypen, “High-Kinetic-Inductance Superconducting Nanowire Resonators for Circuit QED in a Magnetic Field,” *Physical Review Applied*, vol. 5, no. 4, p. 044004, Apr. 2016.
